# Ultrasonic irrigation flows in root canals: effects of ultrasound power and file insertion depth

**DOI:** 10.1038/s41598-024-54611-x

**Published:** 2024-03-04

**Authors:** A. Koulogiannis, A. D. Walmsley, P. Angeli, S. Balabani

**Affiliations:** 1 FluME, Department of Mechanical Engineering, University College London (UCL), London, UK; 2 School of Dentistry, College of Medical and Dental Sciences, University of Birmingham, Birmingham, UK; 3 ThAMes, Department of Chemical Engineering, University College London (UCL), London, UK; 4 Wellcome/EPSRC Centre for Interventional and Surgical Sciences, University College London (UCL), London, UK

**Keywords:** Root canal, Ultrasonic, Irrigation, Biofilm, Ultrasound power, Insertion depth, Particle image velocimetry, Fluid dynamics, Mechanical engineering, Fluid dynamics, Biomedical engineering

## Abstract

Ultrasonic irrigation during root canal treatment can enhance biofilm disruption. The challenge is to improve the fluid flow so that the irrigant reaches areas inaccessible to hand instrumentation. The aim of this study is to experimentally investigate how the flow field and hydrodynamic forces induced by ultrasonic irrigation are influenced by the ultrasound power and file insertion depth. A root canal phantom was 3D printed and used as a mold for the fabrication of a PDMS channel. An ultrasonic instrument with a #15K-file provided the irrigation. The flow field was studied by means of Particle Image Velocimetry (PIV). The time averaged velocity and shear stress distributions were found to vary significantly with ultrasound power. Their maximum values increase sharply for low powers and up to a critical power level. At and above this setting, the flow pattern changes, from the high velocity and shear stress region confined in the vicinity of the tip, to one covering the whole root canal domain. Exceeding this threshold also induces a moderate increase in the maximum velocities and shear stresses. The insertion depth was found to have a smaller effect on the measured velocity and shear stresses. Due to the oscillating nature of the flow, instantaneous maximum velocities and shear stresses can reach much higher values than the mean, especially for high powers. Ultrasonic irrigation will benefit from using a higher power setting as this does produce greater shear stresses near the walls of the root canal leading to the potential for increased biofilm removal.

## Introduction

Bacterial biofilms are assemblies of different species of oral microorganisms that attach to the teeth. If left untreated, they facilitate the disease process and bacteria will move through the tooth structure to reach the root canal where they can further grow leading to infection and inflammation of the pulp^1–5^. The complex anatomy of the root canal is a challenge for biofilm removal. Any antimicrobial irrigants or antibacterial agents will have difficulty accessing those areas where the bacterial colonies are residing^6–9^.

While syringe-based irrigation is commonly employed and well-studied^10–13^, ultrasonic irrigation provides a more effective method of biofilm disruption^14–16^. The advantage of ultrasound is that it will generate both microstreaming and cavitation^17,18^ producing high velocities and shear stresses within the root canal. This allows the irrigant to access parts of the root canal system that are inaccessible to other methods.

Previous research has analyzed both theoretically and experimentally the ultrasound-induced flow field in root canals^19^ and quantified the effect of cavitation^20–23^. The effect of the ultrasonic instrument tip geometry on the fluid dynamics in the root canal has also been studied^24^. Such analytical and experimental findings have been compared to computational results^25^. The ultrasound-induced flow field inside confined geometries such as root canals is complex and highly dependent on the operating parameters, such as ultrasound power and file insertion depth. These parameters may influence the induced irrigation flows and in turn potentially increase biofilm removal. Thus, they provide a means to control and optimize the irrigation process and merit further investigation. To the best of our knowledge there are no systematic experimental studies of the effects of these parameters on the induced flow field and thus the irrigation efficiency.

The aim of the present study is to experimentally characterize ultrasonic irrigation flow fields inside a root canal model as a function of the applied ultrasonic instrument power (which corresponds to the oscillation amplitude of the file), and file insertion depth. Particle Image Velocimetry (PIV) was employed to measure the induced velocity field. This novel approach reports the fluctuating and time-averaged velocity and shear stress distributions for a wide variety of the operating parameters of the ultrasonic instrument.

## Materials and methods

### Root canal model fabrication

An idealized curved root canal geometry was chosen based on data from clinical studies^13^. The canal had a length of 2 cm, a tapered diameter varying from 2 mm coronally to 0.2 mm at the apex and a radius of curvature of 65°. The canal was 3D printed using Polylactic acid (PLA) (Cura S3; Ultimaker B.V., Utrecht, Netherlands) and used as a negative mold to fabricate a channel using polydimethylsiloxane (PDMS) (Sylgard 184; Dow, Midland, MI). The apical end of the channel was sealed.

It should be noted that some simplifications have been made on the characteristics of our model compared to the in vivo root canal in terms of geometry and material properties. Root canals have complex geometries with varying diameters and curvature and typically include features such as lateral canals, deltas and isthmuses^26,27^. These features can affect the flow patterns induced by ultrasound therein. In this work, a more generic and simplified curved geometry was selected to allow us to interpret and generalise the results of this study.

The material properties of the fabricated model differ from those of dentine. For example, dentine is a stiff material with an average Young Modulus of 30 GPa^28^ while PDMS is highly compliant with an average Young Modulus of  around 2 MPa^29,30^. However, the PDMS properties are not expected to affect the measured velocity fields as no wall deformation was observed in any part of the root canal during the experiments.

### Particle image velocimetry (PIV)

**Figure 1:**
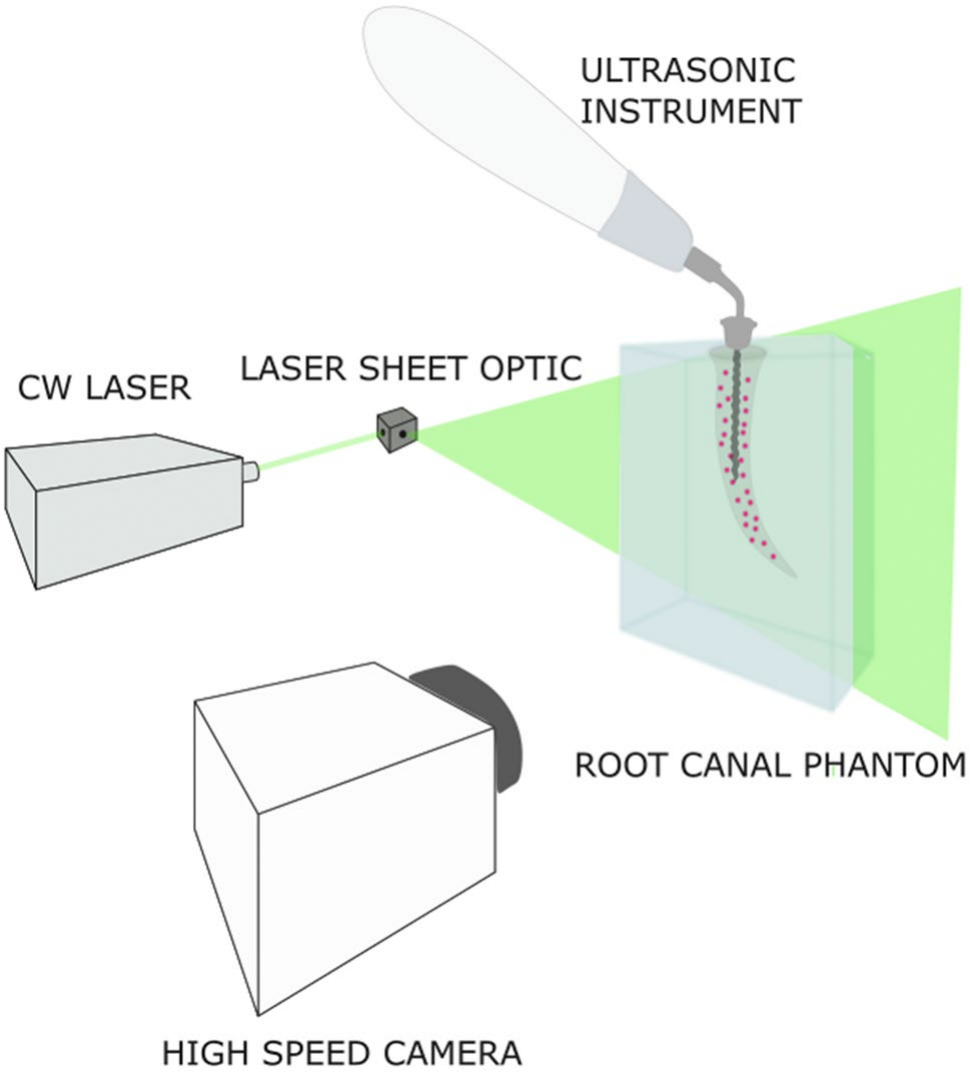
Experimental setup showing root canal model and PIV arrangement.

To measure the velocity field in the ultrasonic irrigation flows under investigation the experimental setup shown in Fig. 1 was used**.**

A 0.425 w/w Sodium Iodide (NaI; Merck KGaA, Darmstadt, Germany)—water solution was used as the working fluid to match the refractive index of PDMS (RI of 1.42). This allowed maintaining the physical properties of the solution closely with those of water^31^. The fluid had a viscosity of 1.67 mPa s and a density of 1.48 g/cm^3^ at 20 °C.

The solution was seeded with 10 μm Nile Red fluorescent particles (PMMA-RhB-FRAK-Particles; microParticles GmbH, Berlin, Germany). The flow in the primary oscillation plane of the file was illuminated using a continuous 532 nm laser (Laserglow Technologies, Toronto, ON, Canada) and imaged using a high-speed camera (Phantom Miro 340; Ametek Inc. Berwyn, PA) operating on a single frame mode and a frame rate of 6 kHz. A compromise between spatial and temporal resolution was made to resolve the flow field in the canal; hence a sampling frequency lower than the tip oscillation was selected which was deemed sufficient to capture the time averaged and fluctuating features of the flow. The latter was verified by resampling the data post processing and recalculating the time averaged and fluctuating quantities.

For each experiment, 500 images were acquired. The image resolution was 1200 × 400 pixels with a pixel size of 10 μm. The acquired images were processed using PIV algorithms implemented in Dynamic Studio (Dantec Dynamics, Skovlunde, Denmark). An adaptive PIV algorithm with a grid step of 8 pixels was used to determine the instantaneous velocity field from which time averaged and fluctuating quantities were extracted. The 2D shear stress distribution was estimated as shown in Eq. (1):

1 $${\tau }_{xy}=\mu \left(\frac{\partial v}{\partial x}+\frac{\partial u}{\partial y}\right)$$

where $\mu$ is the dynamic viscosity of the solution and *u* and *v* the velocity components on the x and y axis respectively. The derivatives were calculated using a central differencing scheme throughout most of the domain, whereas forward or backward differencing was used along the edges of the vector map.

### Ultrasonic irrigation

An ultrasonic instrument (DTE S6; Woodpecker Medical Instrument Co., Ltd., Guilin, China) operating at a frequency of 28 kHz, mounted to an endodontic tip with a #15K-file, was employed. To avoid tip contact with the curved wall of the channel during experimentation, and hence minimize the risk of potential file damage and changes in the induced flow field due to wall impact, the file was inserted only up to the mid-length of the channel (10 mm coronally).

The power setting dial of the ultrasonic instrument allowed increases from 1 to 15 which corresponded to an electric power range of 1.5–19.5 W. The latter was calculated from current and voltage measurements, described in the Supplementary Material, and found to increase exponentially with the power setting of the ultrasonic instrument. The use of electric power was preferred to tip oscillation amplitude due to the difficulties in measuring the latter accurately using the commonly used laser vibrometry. The small diameter of the file in combination with the high velocity of oscillation complicates the focusing of a laser beam on the tip and affects the generated signal, leading to inaccurate measurements.

Tip insertion depth studies were conducted at a fixed power setting of 7 (medium power), corresponding to an electric power of 4.3 W. The insertion depth was varied by gradually withdrawing the tip from its mid length position (10 mm inside the channel) in seven increments of 1 mm, by means of a single-axis micrometer translation stage. Thus, the flow domain examined encompasses the coronal and middle third of the root canal.

## Results

**Figure 2:**
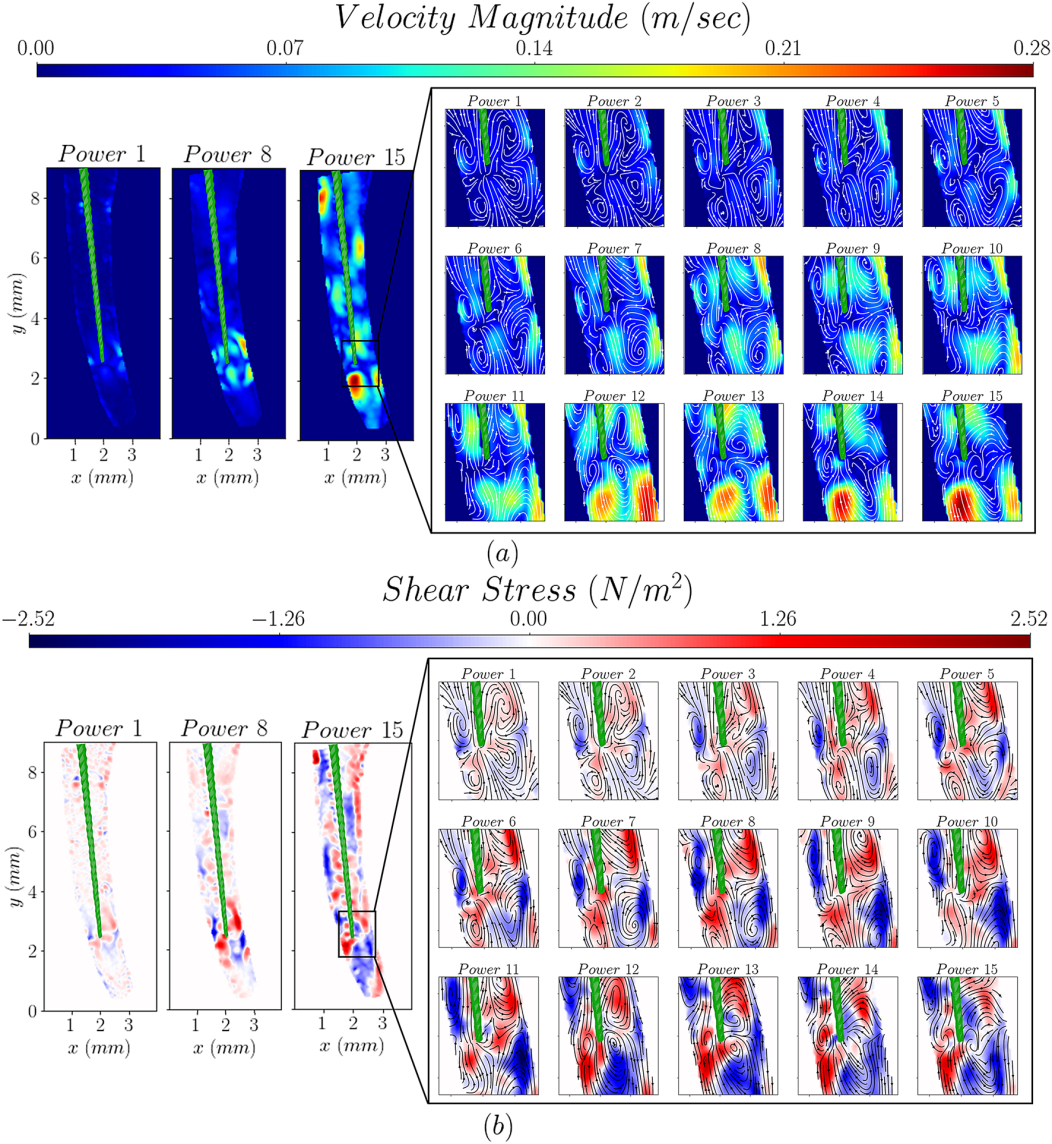
The effect of ultrasound power on the velocity (**a**) and shear stress distributions (**b**). Power numbers indicate the setting used.

**Figure 3:**
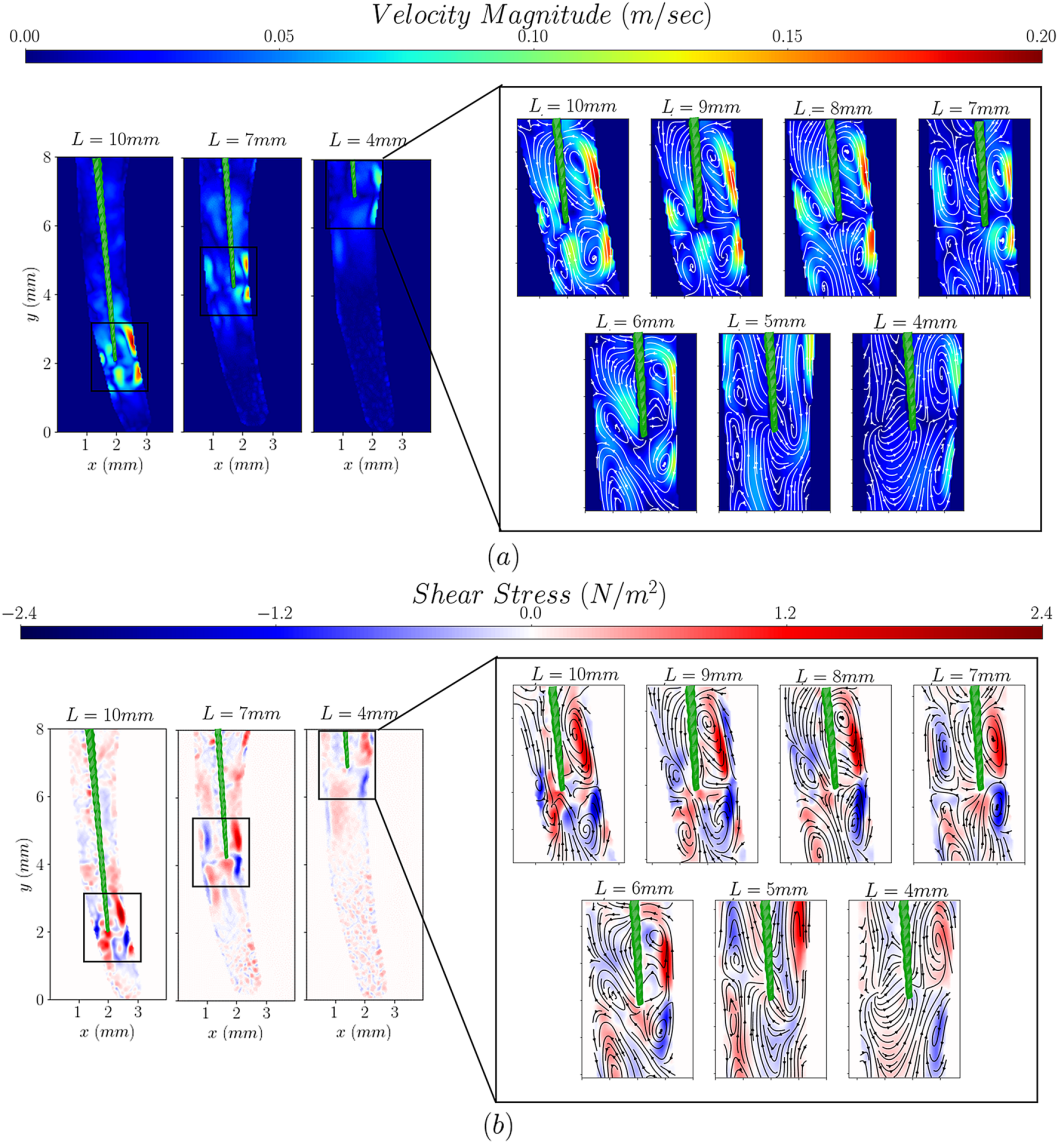
The effect of file insertion depth on the velocity (**a**) and shear stress distributions (**b**). x and y axes are used to guide the eye and L represents the insertion depth measured coronally.

Time-averaged velocity and shear stress magnitude colourmaps, with superimposed flow streamlines, are shown in Figs. 2 and 3 for different ultrasound powers and tip insertion depths, respectively. The flow field of the whole studied domain (coronal and middle third of the root canal) is shown for three selected cases (results for all cases can be found in the Supplementary Material). A more detailed region of interest, around the edge of the tip, is shown for all the powers and tip insertion depths investigated.

The ultrasound power has a significant effect on the flow structure inside the root canal (see Fig. 2). For low power settings, microstreaming effects give rise to two high velocity jets near the edge of the tip. Impingement of these jets onto the canal walls creates four vortical structures which subsequently lead to high velocities and shear stresses near the walls, in the vicinity of the tip edge. As the power increases, these vortical structures gradually merge and the high velocity/shear stress regions expand into the whole domain, particularly near the root canal walls.

The insertion depth does not significantly affect the flow patterns inside the root canal (see Fig. 3) for the insertion range investigated. The four vortical structures remain apparent even for low insertion depths. High velocity and shear stress regions are confined to the vicinity of the tip in all insertion depths studied due to the fixed power setting.

**Figure 4:**
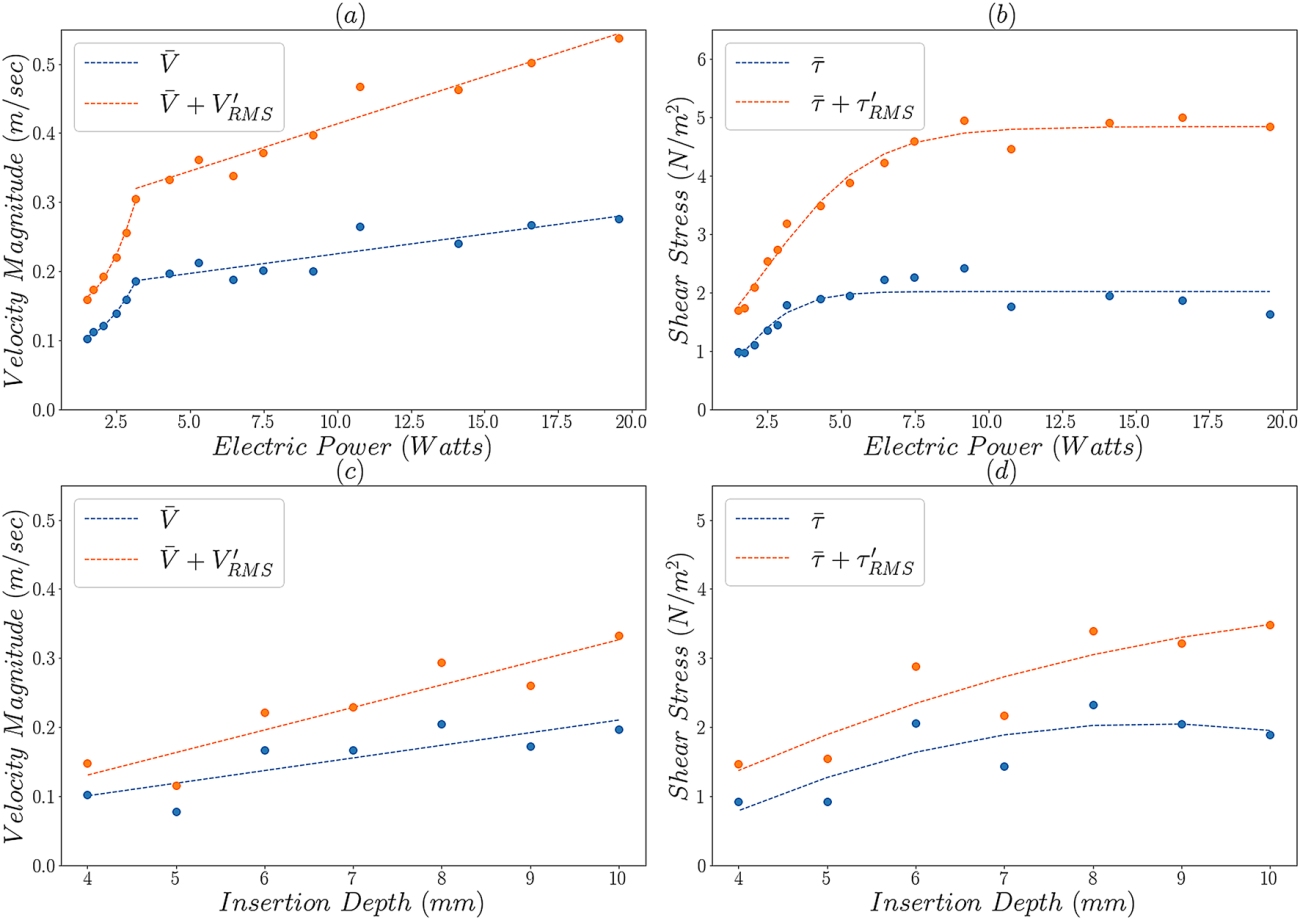
Evolution of the maximum time-averaged and fluctuating velocity and shear stress magnitudes as a function of ultrasound power, for an insertion depth of 10 mm (**a**,**b**) and file insertion depth for an ultrasonic power of 7 (**c**,**d**). Trend lines are plotted only to guide the eye.

The variation of the maximum velocity and shear stress magnitudes in the near-tip region of interest (magnified areas in Figs. 2 and 3) is plotted in Fig. 4 as a function of electric power and insertion depth. Both time averaged and fluctuating components are shown, the latter expressed through the root-mean-square.

A non-monotonic behaviour of the maximum velocities with power is observed (see Fig. 4a). Maximum velocities initially increase exponentially with the power of the instrument up to a power of 3.1 W. After that point a linear trend can be observed; the effect of power on the velocity becomes less pronounced and there is more scatter in the data. The variation of shear stresses with ultrasound power exhibits a different pattern characterised by a logarithmic increase followed by a plateau for powers over 7.5 W. This coincides with the change in the flow distribution within the canal and the presence of high velocity/shear stress regions throughout the domain.

The time-averaged, maximum velocity magnitude ranges from 10 cm/s to 28 cm/s in the ultrasound power range investigated while the instantaneous one ranges from 16 cm/s to 54 cm/s (see Fig. 4a), The respective shear stresses range from 1 N/m^2^ to 1.6 N/m^2^ and 1.7 N/m^2^ to 4.8 N/m^2^ for the time averaged and instantaneous values, respectively (see Fig. 4b).

Low insertion depths lead to less flow confinement and diminish the strength of the vortical structures. This results in a linear decrease of the magnitude of the near-wall velocities and shear stresses. The time-averaged maximum velocity magnitude increased twofold, from 10 cm/s to 20 cm/s, as the insertion depth increased from 4 to 10 mm (see Fig. 4c).This also translates into an increase in the shear stresses from 0.9 N/m^2^ to 1.9 N/m^2^ (see Fig. 4d). The respective instantaneous range is from 15 cm/s to 33 cm/s for the velocity and from 1.5 N/m^2^ to 3.5 N/m^2^ for the shear stress (see Fig. 4c,d).

**Figure 5:**
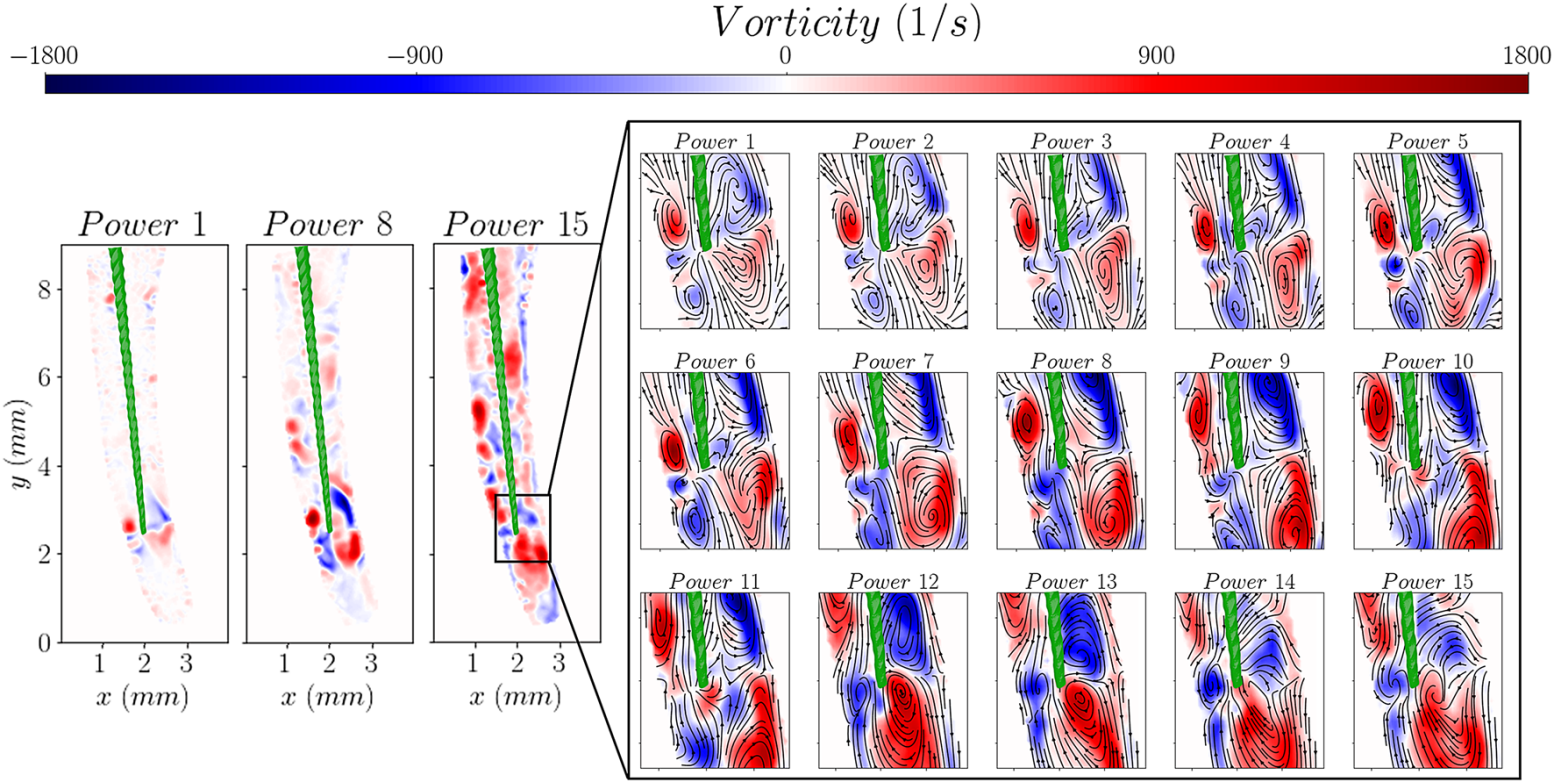
The effect of ultrasound power on the vorticity distribution.

Figure 5 shows the vorticity distribution in the region of interest around the tip of the file as a function of ultrasound power. The four symmetric vortices, arising from the impingement of the streaming jets on the walls of the root canal, can be seen forming around the tip of the file for the lowest power settings (below 2.5 W). They increase in strength with ultrasound power and eventually start merging when the power surpasses the threshold of 7.5 W. The flow becomes progressively more chaotic and at very high power settings, above 17 W, no flow structures are evident.

**Figure 6:**
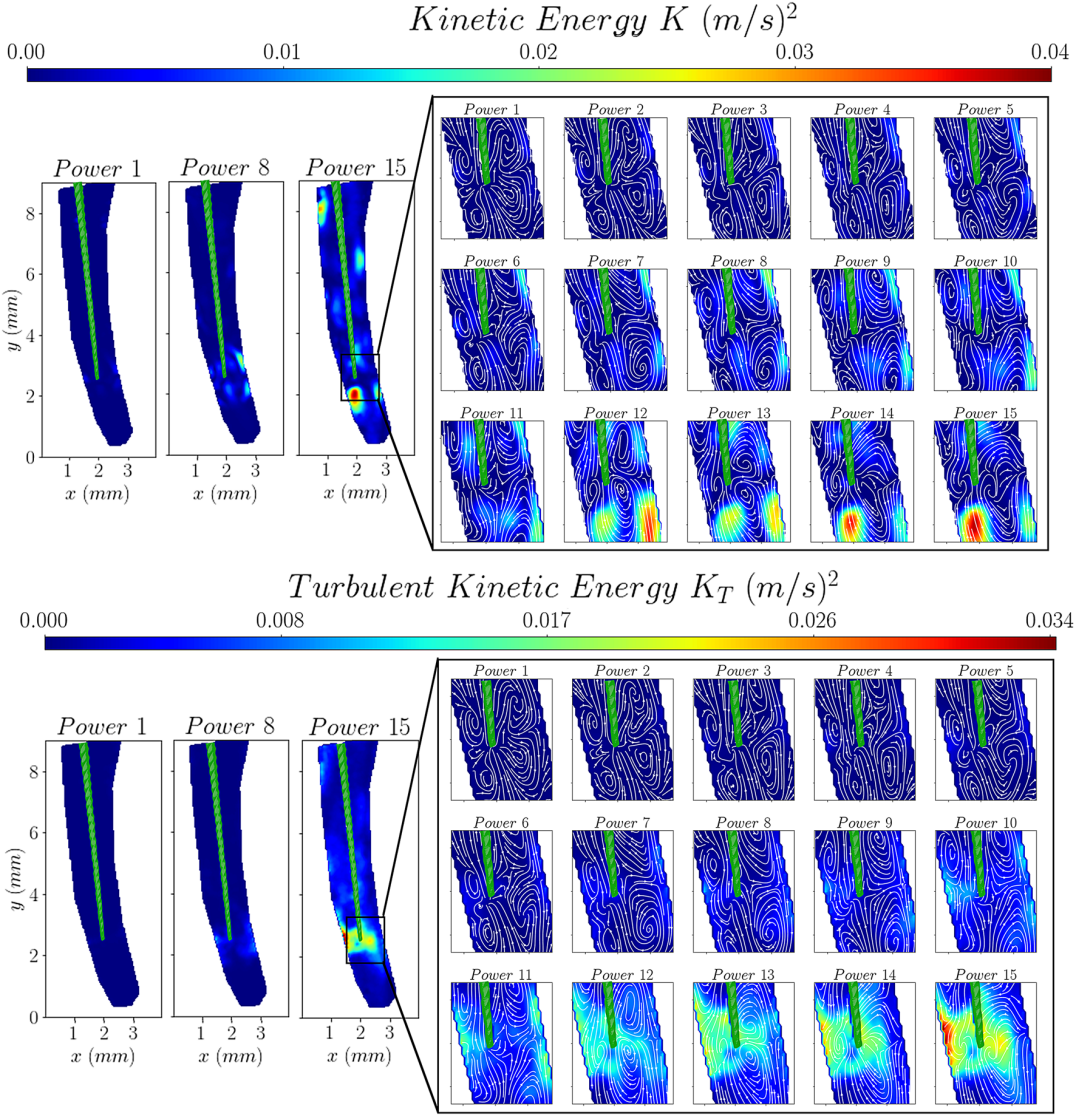
The effect of ultrasound power on the mean and turbulent kinetic energy distributions.

The variation of the mean and turbulent kinetic energy distributions, estimated from the measured velocity components (as shown in Eqs. 2 and 3), with ultrasound power is plotted in Fig. 6. The mean and turbulent kinetic energy are defined as:

2 $$K=\frac{1}{2}\left({u}^{2}+{v}^{2}\right)$$

3 $${K}_{T}=\frac{1}{2}\left(\overline{{({u}^{\prime})}^{2}}+\overline{{({v}^{\prime})}^{2}}\right)$$

The mean flow kinetic energy, *K,* follows the velocity distributions, shown in Fig. 2, as expected. For low powers the maximum kinetic energy is located near the walls where impingement of the jets occurs. As the ultrasound power increases the location of the maximum kinetic energy moves further below the tip of the file.

On the contrary, the distribution of the turbulent kinetic energy, *K*_*T*_, is relatively uniform for low powers, below 7.5 W. However, as the power increases above this power setting a region of high turbulent kinetic energy appears around the tip of the file, due to the increase in fluctuating velocities therein. These result from the higher oscillation amplitude of the tip of the file and they mark the transition of the flow above a certain power threshold.

**Figure 7:**
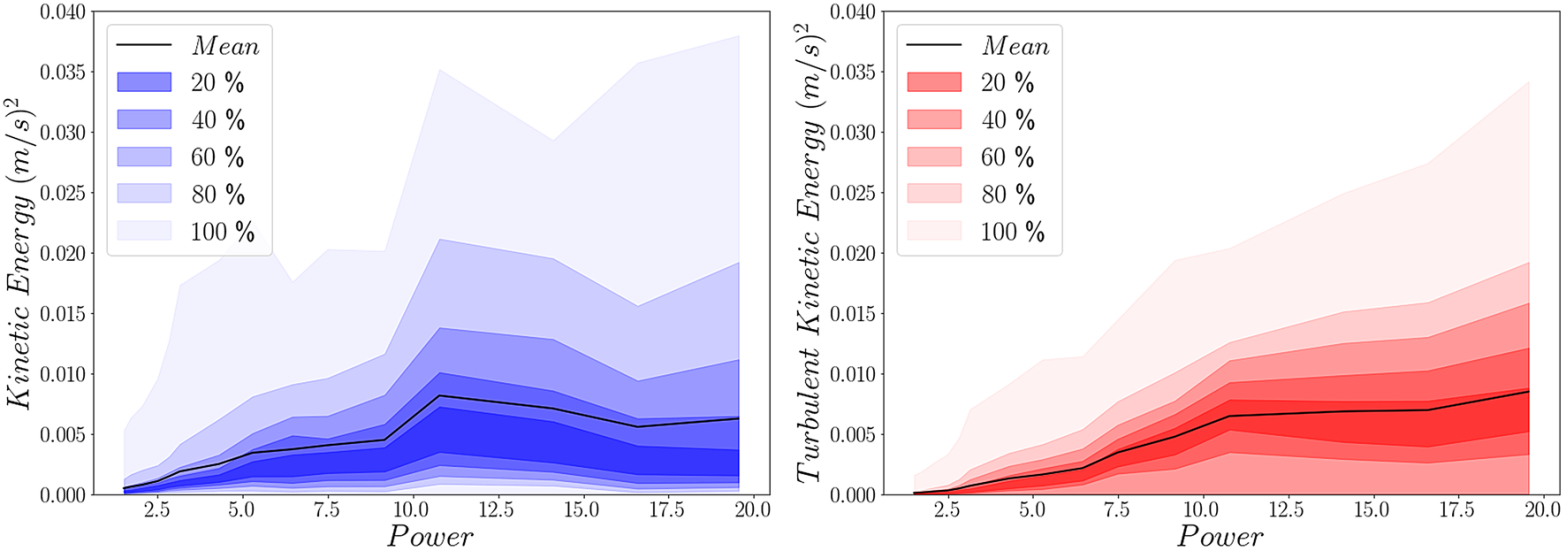
Fan-charts of the mean and turbulent kinetic energy distribution in the region of interest around the tip of the file.

To better illustrate the overall effect of ultrasound power on the kinetic energy of the flow field around the tip, fan-charts of both the mean and turbulent kinetic energy distributions (shown in Fig. 5) are plotted in Fig. 7. These were produced by spatially averaging the values in the region of interest around the tip shown in Fig. 6.

For low power settings, below the aforementioned threshold of 7.5 W, the mean flow kinetic energy surpasses the turbulent one both in terms of maximum and mean values. This is attributed to the high time averaged velocities emerging from the impingement of the jets on the walls of the root canal and relatively low velocity fluctuations associated with these power settings (see Fig. 4). As the power increases the mean flow kinetic energy appears to level off whereas the turbulent kinetic energy increases linearly. This is due to the fact that mean flow kinetic energy values increase locally near the walls for powers above 7.5 W (as shown in Fig. 6) while turbulent kinetic energy throughout the region of interest around the tip of the file.

## Discussion

Instrument power setting plays a significant role in the ultrasound induced flows inside the confined space of a root canal; increasing the power intensifies momentum transport in the root canals. This is reflected in Fig. 4a showing a significant increase in the maximum velocities achieved. Although the range of maximum time-averaged velocities agrees with the values reported in the literature for similar files^17,18^, decomposing the instantaneous velocity field into time averaged and fluctuating parts shows that the velocities can instantaneously reach twice as high values compared to time-averaged ones. This is especially seen at high power settings and is not surprising given the oscillating nature of ultrasound induced flows^25^.

The nature of the flow changes when the electric power surpasses 9.2 W. This is associated with more chaotic flow and higher intensity of fluctuating velocities (see Supplementary Material) which are no longer localized near the tip but spread to the rest of the flow domain.

The change in flow structure is also evident in the distribution of time averaged shear stress values (see Fig. 4b) which reaches a plateau at this power setting. This could be due to less uniform velocity distribution (hence steeper velocity gradients) near the walls of the whole root canal domain at high power settings. Shear stress fluctuations show a significant increase with ultrasound power highlighting the importance of the instantaneous magnitudes compared to the time-averaged ones. The time averaged shear stresses are of the same order of magnitude as those reported in both analytical and experimental studies^13,19^. Although the maximum instantaneous velocity values are one order of magnitude lower than those reported by computational studies^25^ instantaneous values measured near the walls of the channel and in the rest of the domain are in good agreement with literature^25^. This is due to the reported maximum values occurring in regions very close to the tip of the file which cannot be resolved with the current optical flow diagnostics due to reflections. Shear stresses in the magnitude range reported in our work have been shown to effectively remove biofilms of different bacterial species present in the dental root canal^32,33.^ Although higher power settings seem to be beneficial from a fluid dynamics point of view, the impact of such settings on damage or potential breakage of the file due to fatigue or impact with the wall of the root canal, should be considered.

While the insertion depth of the file affects the magnitude of the time averaged and instantaneous velocities and shear stresses due to the confinement arising from the tapered nature of the canal (see Fig. 4c,d), their ratio is not significantly affected. This reinforces the argument that the confinement alone cannot increase velocity fluctuations at a particular power level. However, this also suggests that mid-range power settings can lead to a more stable, but highly localised flow field over time for any insertion depth, potentially leading to a better controlled irrigation process. It should be noted that the observed stability of the flow field is due to the file been able to oscillate freely within the canal space studied. Inserting the file deeper into the apical third of the curved root canal, would result in periodic contact with the wall. Such contact can significantly affect the flow field.

The flow structures and high velocities generated by jet impingement on the walls of the root canal, even for low ultrasound powers, suggest that a better penetration of antibacterial agents inside the dentinal tubules could be achieved with ultrasonic irrigation. The instantaneous flow field can also play an important role in this process as the study highlights. Although the time- averaged flow field can elucidate the underlying flow dynamics of ultrasonic irrigation, it might not be sufficient to determine the potential of irrigant flows for biofilm removal. In some cases, the fluctuating component of the flow can become equally important with the time-averaged flow.

While the ultrasound induced flow field is three dimensional, velocities were measured on one plane and hence a two dimensional approximation was used in the calculation of the velocity magnitude and shear stresses. This could potentially lead to an underestimation of the velocity magnitude and shear stress distributions.

It should be noted, that while cavitation is highly associated with ultrasound induced flows, and has been reported in published works^21–36^, in the present study cavitation was only observed in the free surface, i.e. in the boundary of the flow domain, and thus far from the region of interest. No cavitation was evident in the rest of the domain for any of the studied cases. This can be attributed to a number of factors, primarily the fluid and the type and size of the file tip employed. Literature suggests that the properties of the irrigant seem to have a significant effect on the generation of cavitation^35,36^. Water results in significantly lower cavitation compared to other commonly used irrigants such as NaOCl. The tapering of K-files results in lower cavitation compared to untapered IrisSafe files. Furthermore, the size of the file can be crucial as larger diameter files can generate more cavitation bubbles^21,34^.

All studies agree that the combination of K15/25 files with water (used in the present study) produce very small amount of cavitation bubbles that are only present either very close to the file or on the coronal part where there is an interface of water with air. No cavitation has been reported near the walls of the root canal, independent of variations of the file/wall distance^34^.

## Conclusions

The ultrasound induced flow field inside a root canal is dependent on both the power and the insertion depth of the tip. The findings suggest that, high shear stresses near the walls, and thus successful biofilm removal, can be achieved on the whole domain of the root canal by optimising the operating parameters and hence the irrigation process. Further work is needed to translate these findings to improved clinical debridement of the root canal.

Future studies will be focused on a higher temporal and spatial resolution analysis of the flow close to the root canal walls as well as inside dentinal tubule models in order to fully quantify the ability of ultrasound to penetrate inaccessible areas within the root canal anatomy.

### Supplementary Information

Supplementary Information.

## Data Availability

The datasets obtained and analyzed in the present study are available from the corresponding author upon request.
