# Supplementary material for: Ultrasonic irrigation flows in root canals: effects of ultrasound power and file insertion depth
Source: Sci Rep. 2024 Mar 4;14:5368. doi: 10.1038/s41598-024-54611-x (PMC10912427; doi:10.1038/s41598-024-54611-x)
Supplement: Supplementary file 1 — Supplementary Information. [file 41598_2024_54611_MOESM1_ESM.pdf]

Supplementary Material

Electric Power Measurement

A 1 Ohm resistor was fitted in the return path of the piezoelectric transducer to enable measurements of the electric power of the ultrasonic instrument. A voltmeter was then used to measure the voltage (V) driving the transducer (including the 1 Ohm resistor) and the voltage across the 1 Ohm resistor which was then translated to current (mA). A Fast Fourier Transform (FFT) was used to denoise both current and voltage measurements and obtain the amplitudes (see **Figure S1a** and **FigureS1b**). The electric power (W) was calculated by multiplication of the measured current and voltage values (see **Table S1** and **Figure S1c**).

Power Setting	Voltage (V)	Current (mA)	Power (W)
1	41.61	36.2	1.5
2	44.61	38.2	1.7
3	47.0	43.5	2.0
4	54.8	45.6	2.5
5	57.9	48.8	2.8
6	61.2	51.4	3.1
7	74.6	57.6	4.3
8	83.6	63.1	53.
9	95.1	67.8	6.5
10	102.4	73.1	7.5
11	112.8	81.2	9.2
12	120.8	89.2	10.8
13	137.7	102.5	14.1
14	148.5	11.7	16.6
15	159.0	123.0	19.6

Table S1: Voltage, Current and Power measurements for each power setting of the ultrasonic instrument.

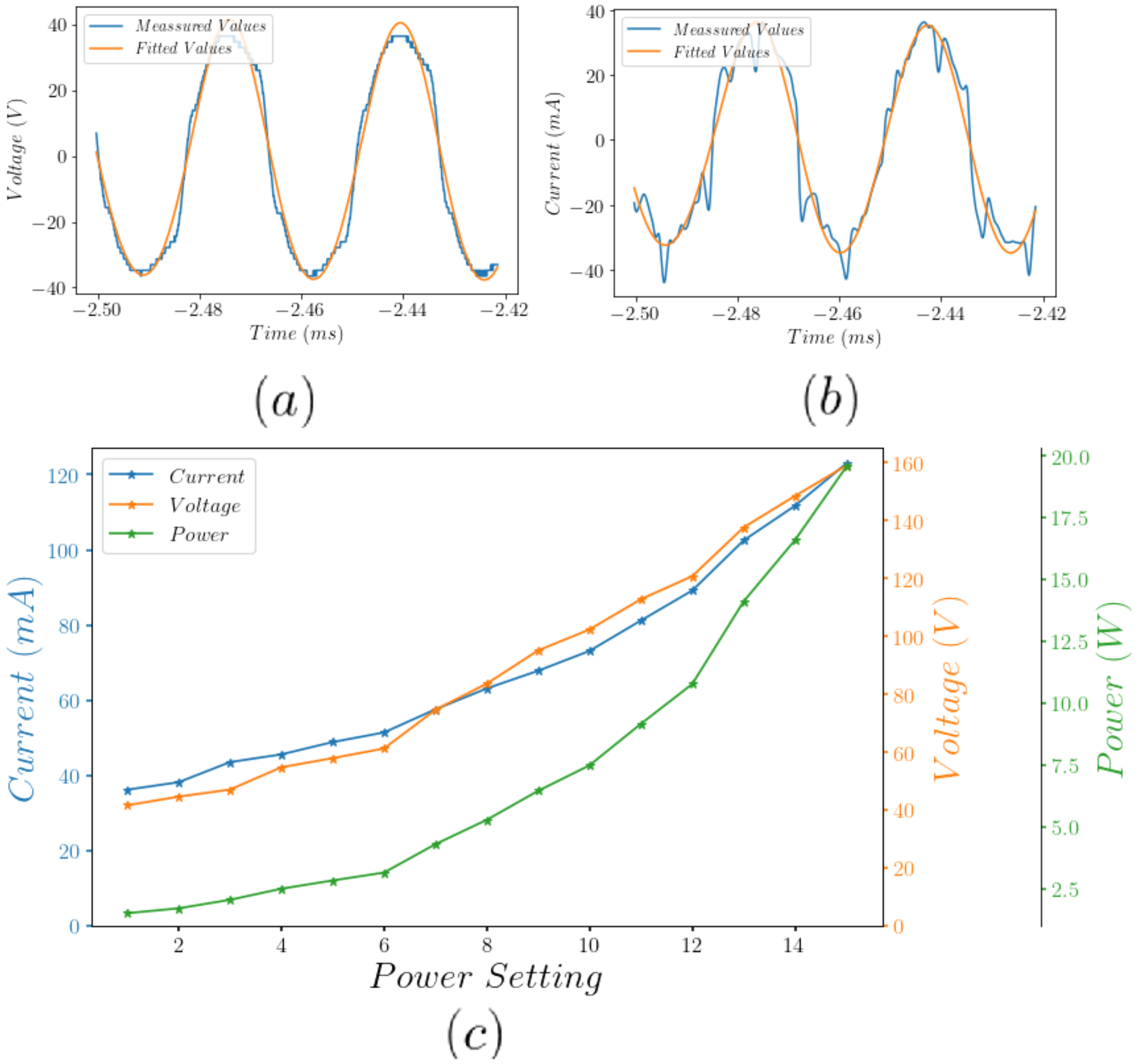

Figure S2: Raw and fitted data for voltage (a) and current (b) measurements; voltage, current and power values as a function of the power setting of the ultrasonic instrument (c) .

Flow fields measured by PIV for all power settings

Due to brevity only selected flow fields were shown in the manuscript. This section shows all the measured flow fields, the velocity and shear stress distributions on the whole root canal domain is shown in **Figures S2 and S3** (Equivalent of Figure 2 and Figure 3 in the main text).

The data used to generate the cumulative results in Figure 4 of the main text are shown in **Table S2 and Table S3**.

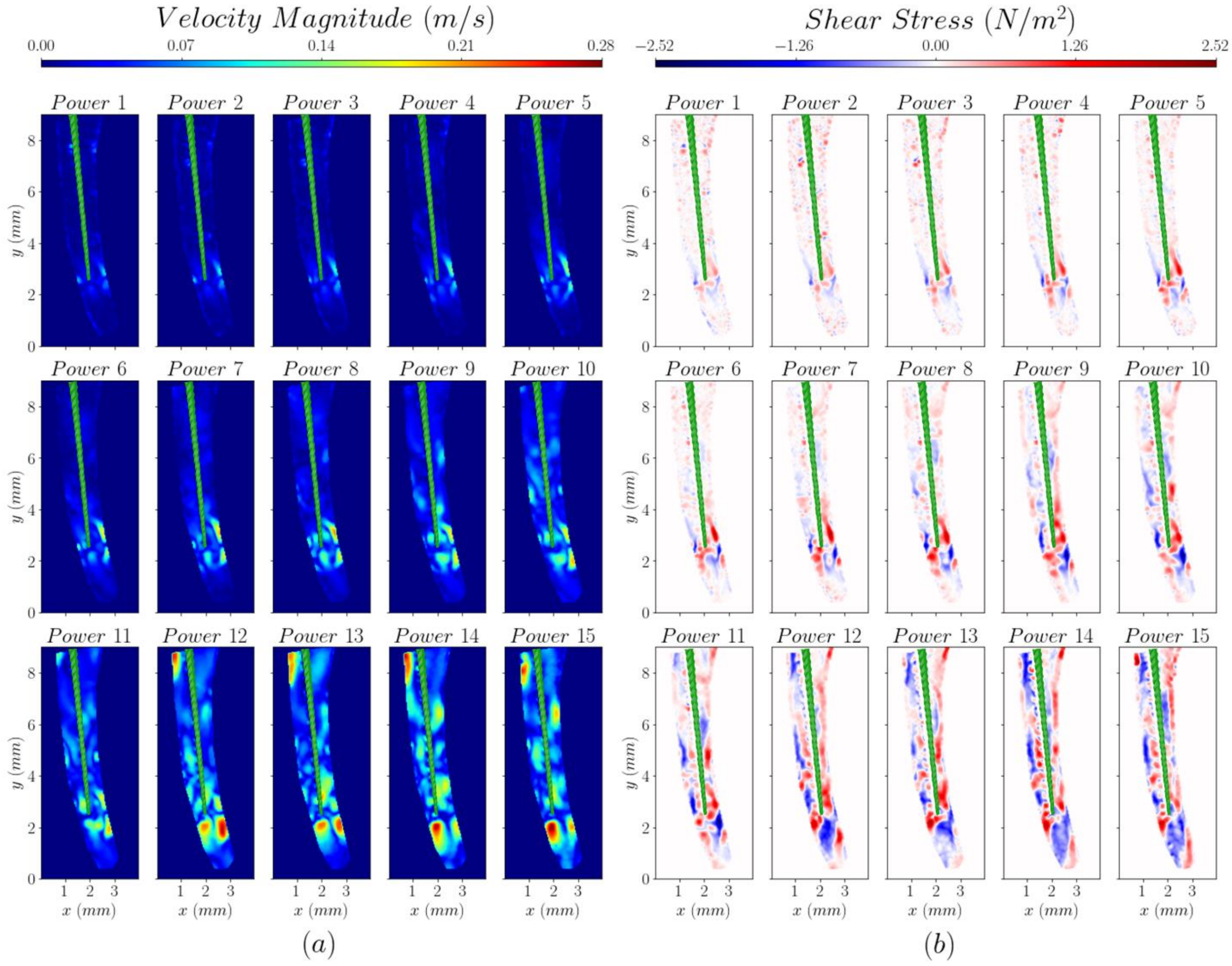

Figure S3: The effect of ultrasound power on the velocity (a) and shear stress distributions (b).

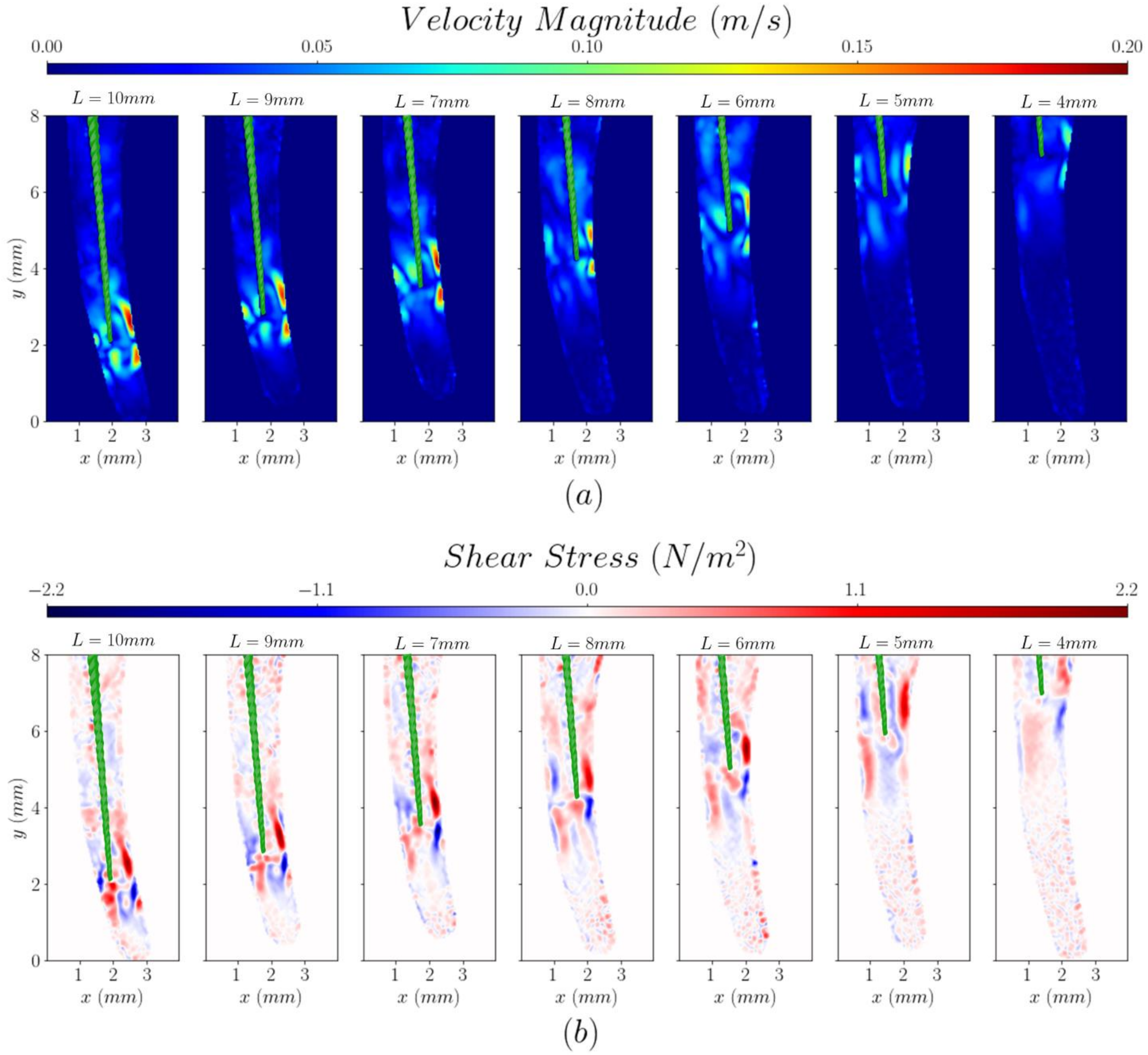

Figure S4: The effect of file insertion depth on the velocity (a) and shear stress distributions (b) .

Insertion Depth (mm)	Mean Velocity (m/s)	RMS Velocity (m/s)	Mean shear stress (N/m^2)	RMS shear stress (N/m^2)
4	0.197	0.197	1.893	1.586
5	0.173	0.173	2.045	1.166
6	0.205	0.205	2.327	1.069
7	0.167	0.167	1.432	0.741
8	0.167	0.167	2.059	0.827
9	0.078	0.078	0.925	0.622
10	0.102	0.102	0.921	0.548

Table S2: Evolution of the time-averaged and fluctuating maximum velocity and shear stress magnitudes as a function of the file insertion depth for an ultrasonic power setting of 7.

Power Setting	Mean Velocity (m/s)	RMS Velocity (m/s)	Mean shear stress (N/m^2)	RMS shear stress (N/m^2)
1	0.103	0.056	0.990	0.701
2	0.112	0.061	0.970	0.757
3	0.121	0.071	1.108	0.980
4	0.138	0.081	1.358	1.177
5	0.159	0.097	1.446	1.292
6	0.186	0.118	1.786	1.401
7	0.197	0.135	1.893	1.586
8	0.213	0.149	1.947	1.926
9	0.188	0.151	2.224	1.995
10	0.201	0.170	2.256	2.333
11	0.201	0.197	2.423	2.523
12	0.265	0.202	1.766	2.695
13	0.241	0.223	1.947	2.953
14	0.267	0.234	1.868	3.126
15	0.276	0.261	1.625	3.215

Table 3: Evolution of the time-averaged and fluctuating maximum velocity and shear stress magnitudes as a function of ultrasound power, for an insertion depth of 10 mm.
